# Dietary Protein Requirement of Juvenile Dotted Gizzard Shad *Konosirus punctatus* Based on the Variation of Fish Meal

**DOI:** 10.3390/ani13050788

**Published:** 2023-02-22

**Authors:** Tao Liu, Xinzhi Weng, Jiteng Wang, Tao Han, Yuebin Wang, Xuejun Chai

**Affiliations:** 1 Department of Aquaculture, School of Fisheries of Zhejiang, Ocean University, Zhoushan 316022, China; 2 Qiandongnan Agriculture Science Institute, Qiandongnan 556000, China; 3 Aquatic Technology Extension Station of Taizhou, Taizhou 318000, China; 4 Zhejiang Marine Fisheries Research Institute, Zhoushan 316021, China

**Keywords:** *Konosirus punctatus*, growth, feed utilization, body composition, energy retention, dietary protein levels

## Abstract

**Simple Summary:**

The dotted gizzard shad *Konosirus punctatus* is a typical representative of small commercial fish. However, the dietary protein requirement of this species is still unknown, which has brought difficulties to culture and reproduction as well as artificial release. Fish meal has been the preferred protein source for aquafeeds. For this reason, fish meal was used as the single protein source in this study. Juvenile dotted gizzard shad were fed diets with different protein levels based on the variation of fish meal and were tested for growth, survival, feed utilization, body composition, digestive enzymes, and immunity, especially on the utilization of protein from the perspective of energy retention. The results of this study demonstrated that appropriate protein levels could improve growth and feed utilization of dotted gizzard shad. Excessive dietary protein inclusion alternates the digestive enzyme activities and metabolism of amino acids. The research outcomes will have good commercial potential for the development of formula feed for dotted gizzard shad.

**Abstract:**

An 8-week feeding trial was conducted to investigate the effects of dietary protein levels on growth performance, feed utilization, and energy retention of juvenile dotted gizzard shad *Konosirus punctatus* based on the variation of fish meal. Fish meal was used as the sole protein source; five semi-purified diets were formulated with varying crude protein (CP) levels of 22.52%, 28.69%, 34.85%, 38.84%, 45.78% (CP1-CP5 diets). A total of 300 uniform juveniles with initial body weight 3.61 ± 0.20 g fish^−1^ were randomly divided into five groups with three replicates in each group. The results showed that different CP levels did not significantly affect the survival of juvenile *K. punctatus* (*p* > 0.05). The values of weight gain (WG) and specific growth ratio (SGR) showed a general enhancing trend and then weakened with increasing dietary CP levels (*p* > 0.05). Feed utilization also improved with increasing dietary CP levels (*p* > 0.05), and the optimal feed conversion ratio (FCR) value was found in fish fed the diet with CP3 (*p* > 0.05). The rise of dietary CP from 22.52% to 45.78% enhanced the daily feed intake (DFI) and protein efficiency ratio (PER) values of *K. punctatus* (*p* < 0.05). With the increase of dietary CP levels, daily nitrogen intake (DNI), energy retention (ER), and lipid retention (LR) elevated, while retention (NR), daily energy intake (DEI), and daily lipid intake (DLI) reduced (*p* < 0.05). No statistical differences in the content of water, crude protein, and crude lipid were observed among different treatments (*p* > 0.05). The activity of lipase in CP3 and CP4 diets was significantly higher than that of the CP1 diet (*p* < 0.05). Fish fed CP2 and CP3 diets had significantly higher amylase activity than that of the CP5 diet (*p* < 0.05). The levels of alanine aminotransferase (GPT) first enhanced and then decreased as dietary CP levels raised. The second-order polynomial regression model analysis of the WG and FCR indicated that the optimal dietary protein level for *K. punctatus* is about 31.75–33.82% based on the variation of fish meal.

## 1. Introduction

Protein, as the main organic matter in fish tissue, accounts for about 65–75% of the dry weight [1]. Dietary protein is identified as a crucial source of amino acids for fish synthetic body protein [2] and is an important component of many active substances such as enzymes and hormones with key functions [1,3]. Moreover, despite the fact that dietary protein is the most expensive component of most aquafeeds, it is the essential nutrient to determine the growth rate of fish [4,5]. It has been reported that inadequate dietary protein may hinder growth and even damage the health of fish [6,7,8]. In contrast, excessive dietary protein not only increases feed costs but also results in nitrogen loss and water pollution [9].

Fish meal (FM) has been recognized as the preferred protein source because of its well-balanced nutrition and reasonable palatability [10,11,12]. It is feasible to use fish meal as the only source of protein in feed formulation to determine the optimal dietary protein requirement of fish. For instance, Zhang et al. [4] observed that dietary protein level had a clear effect on growth performance, and the optimum protein level in diets for juvenile *sparus macrocephalus*, defined by the SGR, was 41.4% when white fish meal was the sole protein source and the dietary energy value was 15.9 kJ/g. The optimal dietary protein requirement based on the variation of fish meal for the growth of juvenile *Bidyanus bidyanus* was estimated to be 42.15% [2]. Therefore, it is essential to determine the variation of fish meal that provides the optimum balance of protein requirements, nutrition, and low cost while being environmentally friendly.

The dotted gizzard shad *Konosirus punctatus* is a small nearshore pelagic fish in the family Clupeidae (Clupeiformes), and is widely distributed in the coastal areas of China, South Korea, and Japan [13,14,15]. It is a euryhalinous species and can even live in freshwater [16]. With many advantages such as fast sexual maturity, delicious meat, high market value, and nice appearance, *K. punctatus* has become one of the important commercial aquaculture species in China [15,17,18]. In recent years, due to the decline of marine ecological resources, small, excellent commercial fish are gradually replacing traditional commercial fish, and *K. punctatus* is a typical representative of small commercial fish [19]. To date, the studies on the *K. punctatus* mainly focus on biological characteristics, genetics, and resources [15,17,18,19,20,21]. In nutrition research on fish, ensuring the dietary protein requirement is the most crucial consideration. However, to our knowledge, limited information is utilized about the protein demand in diets of this species, which has brought difficulties to the culture and reproduction as well as artificial release. Therefore, five kinds of semi-purified feeds with different CP levels (ranging from 22.52% to 45.78%) were designed with fish meal as the only protein source in this study to evaluate the effects of different protein levels on the growth performance, feed utilization and energy retention based on the variation of fish meal.

## 2. Materials and Methods

### 2.1. Experimental Diets

Fish meal was used as the only protein source, and fish oil and soybean oil were used as the main lipid sources. Five isoenergetic test diets were prepared to contain graded levels of crude protein (22.52%, 28.69%, 34.85%, 38.84%, and 45.78%, CP1-5 diets) based on the variation of fish meal. The ingredients and proximate composition of diets are shown in Table 1. All dry ingredients were weighed after passing through an ultra-fine pulverizer (120 μm), and fully mixed in a mixer for 20 min. Then, the weighed fish oil and soybean oil and a suitable amount of double-distilled water were added into the mixer until completely mixed. Five kinds of wet pellet feeds (1.5 mm) were individually obtained by a twin-screw extruder (G-250; Technology Machinery Factory of South China University, Guangzhou, China), and were dried at 45 °C. Then, these pellets were sieved and placed in a refrigerator at −20 °C. 

### 2.2. Experimental Fish and Feeding Trial

*K. punctatus* juveniles for the experiments were provided by the Key Laboratory of Mariculture and Improvement, Zhejiang Institute of Marine Fisheries, Zhoushan, China, and the feeding experiment was carried out in this place. Prior to starting the experiments, all fish were placed in concrete ponds (13.0 × 4.0 × 1.5 m; L × W × H) for 2 weeks and fed the same kind of commercial feed. At the beginning of this trial, 20 juvenile fish (initial body weight 3.61 ± 0.20 g fish^−1^) after being fasted for 24 h were randomly selected and put into 15 tanks (300 L). Three replicate tanks were used in order to test 5 kinds of diets for 8 weeks. During feeding, all the test fish were fed with experimental diets at 09:00 and 17:00 to apparent satiation in a natural photoperiod. Sand-filtered and flowing seawater was provided during the culture experiment, and the water flow was maintained at 1 L min^−1^. The roots blower was used for uninterrupted aeration to ensure that dissolved oxygen exceeded 6 mg L^−1^. The temperature, salinity, and pH value of water fluctuated around 27.7 ± 1.4 °C, 26.10 ± 0.9 g L^−1^, and 7.5 ± 0.1, respectively. Ammonia nitrogen concentration was controlled below 0.05 mg L^−1^.

### 2.3. Sample Collection and Analysis

After the acclimatization period, 20 fish with body weight of 3.61 g fish^−1^ were randomly selected and euthanized, and then placed in pre-marked sealed bags and stored in a −80 °C refrigerator for subsequent analysis. After the mariculture experiment, all surviving fish were fasted for 24 h, and the number and weight of the fish in each tank were recorded, respectively. The surviving juveniles in each tank were anesthetized with 150 mg L^−1^ of MS-222. Then, 6 fish were randomly selected from each tank to calculate the morphological parameters including body weight and body length. The 6 fish were dissected to obtain the liver and placed in a −80 °C refrigerator for subsequent physiological index analysis. The other 5 fish were randomly collected from each tank and placed in marked, sealed bags, and stored in a −80 °C refrigerator for subsequent analysis.

Proximate composition of samples (experimental diets, fish) was measured according to AOAC [22]. Moisture was analyzed via a constant temperature drying oven (diets, 105 °C) or a lyophilizer (whole-body, −110 °C, LL1500, Thermo Fisher Scientific, Waltham, MA, USA). An automatic Kjeldahl system (K355/K437 Buchi, Flawil, Switzerland) was used to estimate the crude protein (N × 6.25) of samples based on the Kieldahl method. Soxhlet apparatus (E816, Buchi, Flawil, Switzerland) was used to estimate crude lipid via petroleum ether extraction. A Muffle furnace (550 °C) was used to measure the ash of samples. An adiabatic bomb calorimeter (HWR-15E, Shangli, Shanghai, China) was used to estimate the gross energy of samples.

Lipase and amylase activities of liver were analyzed by the method of Li et al. [23]. Glutamyl pyruvic transaminase (GPT) activity was determined as described by Reitman and Frankel [24]. All parameters were assayed in triplicate by commercial kits (Jiancheng, Ltd., Nanjing, China), and performed by a microplate reader.

### 2.4. Calculation and Statistical Analysis

Initial body weight = IBW; final body weight = FBW.

Weight gain (WG, %) = 100 × (FBW − IBW)/IBW.

Specific growth rate (SGR, % per day) = 100 × (Ln (FBW) − Ln (IBW)/days.

Daily feed intake (g 100 g fish^−1^ day^−1^) = 100 × feed offered/average total weight/days.

Feed conversion ratio (FCR, %) = dry feed consumed/wet weight gain,

Protein efficiency ratio (PER) = wet weight gain/protein intake.

Average body weight (ABW, g) = (IBW + FBW)/2.

Daily nitrogen intake (DNI, g kg ABW^−1^ day^−1^) = feed intake nitrogen/ABW × days.

Daily nitrogen gain (DNG, g kg ABW^−1^ day^−1^) = (FBW × final body nitrogen − IBW × initial body nitrogen)/ABW × days.

Nitrogen retention (NR, %) = 100 × DNG/DNI.

Daily energy intake (DEI, 10^2^ kJ kg ABW^−1^ day^−1^) = feed intake energy/ABW × days.

Daily energy gain (DEG, 10^2^ kJ kg ABW^−1^ day^−1^) = (FBW× final body energy − IBW × initial body energy)/ABW × days.

Energy retention (ER, %) = 100 × DNG/DNI.

Daily lipid intake (DLI, g kg ABW^−1^ day^−1^) = feed intake lipid/ABW × days.

Daily lipid gain (DLG, g kg ABW^−1^ day^−1^) = (FBW × final body lipid − IBW × initial body lipid)/ABW × days.

Lipid retention (LR, %) = 100 × DLG/DLI.

Data were expressed as an average value (*n* = 3) ± SD and analyzed statistically using SPSS 24.0 (IBM, Chicago, IL, USA). Data were analyzed using one-way ANOVA and deviating replicates through Duncan’s multiple-range tests. *p* < 0.05 was regarded as a significant difference. The second-order polynomial regression model was applied to measure the dietary protein requirement for this species on the basis of weight gain (WG) and feed conversion ratio (FCR).

## 3. Results

### 3.1. Growth Performance and Feed Utilization

**Figure 1:**
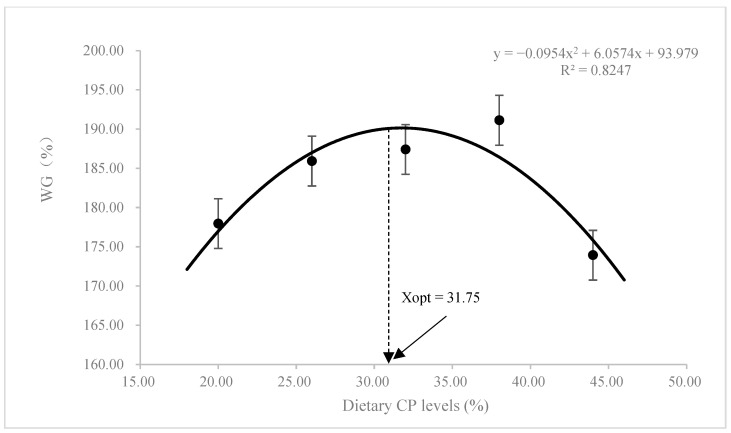
Relationship of weight gain (WG) with dietary crude protein (CP) levels of juvenile *K. punctatus*.

**Figure 2:**
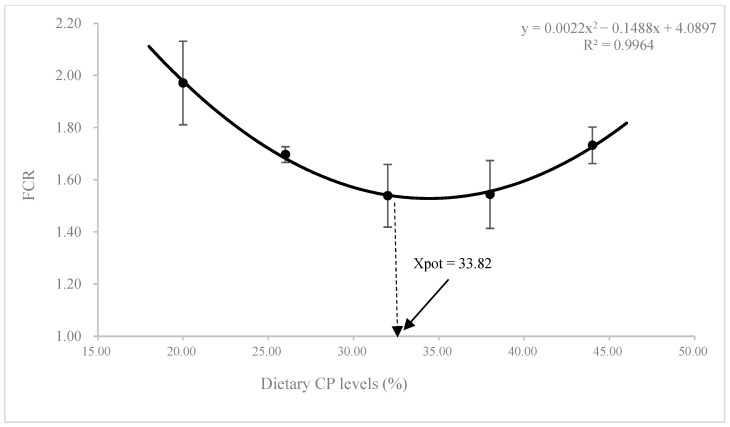
Relationship of feed conversion rate (FCR) with dietary CP levels of juvenile *K. punctatus*.

No statistical differences were observed in the survival rate among different CP levels (*p* > 0.05; Table 2). With the increase of dietary CP levels, the values of weight gain (WG) and specific growth rate (SGR) showed a general enhancing trend and then reduced slightly (*p* > 0.05). Feed conversion efficiency (FCR) was improved by dietary CP levels up to 34.85% and then weakened beyond this level (*p* > 0.05). The values of daily feed intake (DFI) and protein efficiency ratio (PER) in the CP1 diet were the highest, which was significantly higher than those in CP3, CP4, and CP5 diets (*p* < 0.05). According to the second-order polynomial regression model analysis of WG and FCR, the maximal protein requirement for juvenile *K. punctatus* is 31.75–33.82% (Figure 1 and Figure 2).

### 3.2. Retention and Deposition of Energy, Nitrogen, and Lipid

As shown in Table 3, there were no significant differences in the values of daily nitrogen gain (DNG), daily energy gain (DEG), and daily lipid gain (DLG) among different treatments (*p* > 0.05). The values of daily nitrogen intake (DNI), energy retention (ER), and lipid retention (LR) values increased significantly with increasing dietary CP levels (*p* < 0.05). Increasing dietary CP levels from 22.52 to 45.75% reduced daily energy intake (DEI), daily lipid intake (DLI), and nitrogen retention (NR) values (*p* < 0.05).

### 3.3. The Whole-Body Composition

In this study, there were no significant differences in the contents of moisture, crude protein, and crude lipid among dietary CP treatments (*p* > 0.05; Table 4).

### 3.4. Liver Enzyme Activities

**Figure 3:**
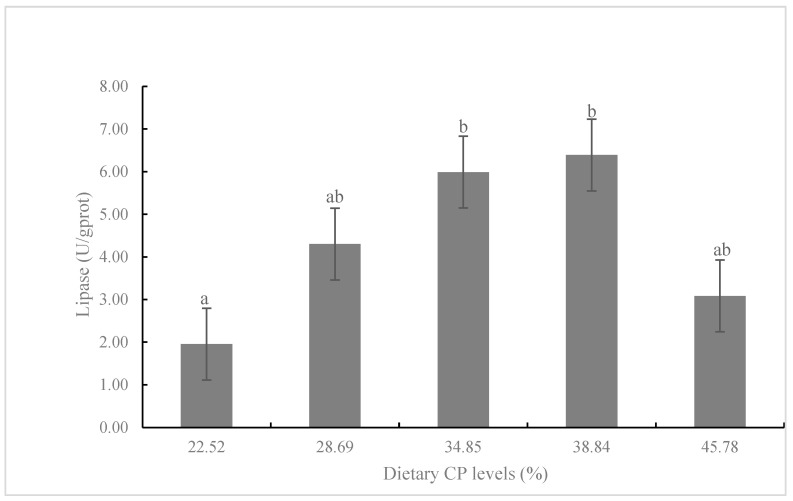
Lipase activity of juvenile *K. punctatus* fed diets containing different CP levels. Value columns with different letters mean significant difference (*p* < 0.05).

**Figure 4:**
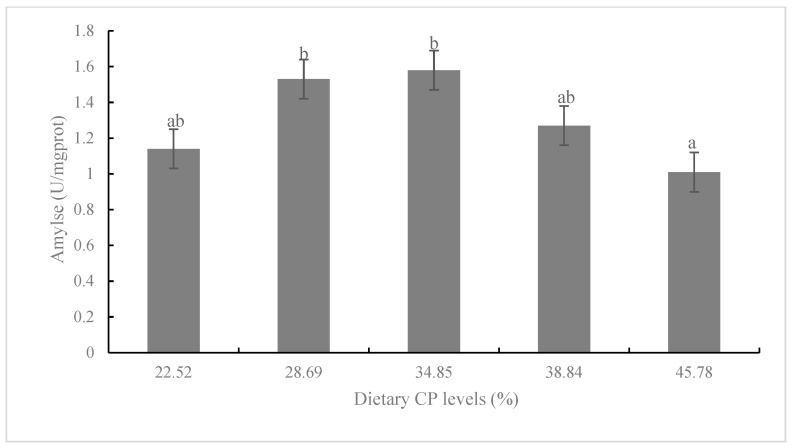
Amylase activity of juvenile *K. punctatus* fed diets containing different CP levels. Value columns with different letters mean significant difference (*p* < 0.05).

**Figure 5:**
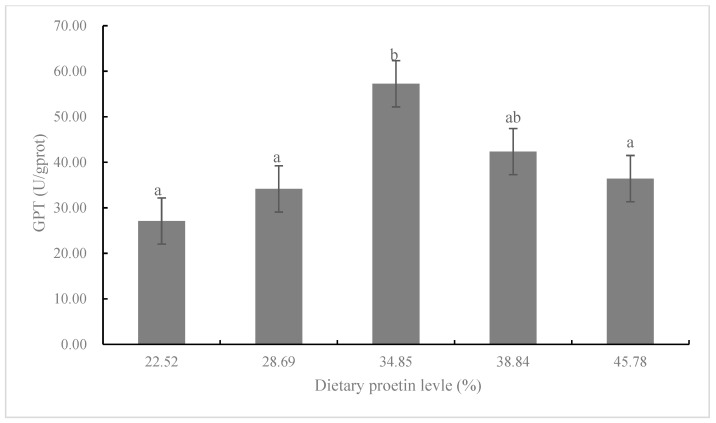
Glutamic-pyruvic transaminase (GPT) activity of juvenile *K. punctatus* fed diets containing different CP levels. Value columns with different letters mean significant difference (*p* < 0.05).

The activity of lipase in CP3 and CP4 diets was significantly higher than that in the CP1 diet (*p* < 0.05; Figure 3). There was no significant difference in the amylase activity among CP1-CP4 diets, but the amylase activity in the CP2 and CP3 diets was significantly higher than that in the CP5 diet (*p* < 0.05; Figure 4). Fish fed the diet with CP3 showed significantly higher glutamyl pyruvic transaminase (GPT) than that in CP1, CP2, and CP5 diets (*p* < 0.05; Figure 5).

## 4. Discussion

During the feeding trial, all experimental diets were properly accepted by juvenile dotted gizzard shad. The survival rate of this species ranged from 92.42% to 96.97%, and no mortalities were ascribed to dietary CP treatments. After 56 days of culture experimentation, although there were no statistical differences in WG and SGR of this species among treatments, WG and SGR showed an overall upward trend and then decreased slowly as dietary CP levels increased when fish meal was used as the single protein source (Figure 1). A similar phenomenon has been observed in the studies of *Puntius gonionotus* [25], *Diplodus vulgaris* [26], *Takifugu rubripes* [27], *Ctenopharyngodon idella* [28], and *Epinephelus akaara* [7]. In addition, the feed utilization of fish fed the low-protein diet was obviously poor, and the highest FCR value appeared in the CP1 diet. Similar results were also observed in other fish species, such as *T. rubripes* [27], *Nibea japonica* [29], and *Lepomis macrochirus* [6]. The feed utilization improved with increasing dietary CP levels, and the optimal FCR was found in fish fed the diet with CP3. However, the FCR in high-protein diets increased again with the further increase of dietary CP levels (CP4-CP5). Based on the second-order polynomial regression model analysis of WG and FCR, the maximum protein requirement for juvenile dotted gizzard shad is about 31.75–33.82% when fish meal was used as the sole protein (Figure 1 and Figure 2). This level is similar to the protein requirement reported for other omnivorous fish, such as 30% *Calrias batrachus* [30], 32% *Zacco barbata* [31], 36% *D. vulgaris* [26]), 30% *Hypostomus commersoni* [32], 30–33% *Betta splendens* Regan [33], and 30 % *Chanos chanos* [8].

Generally, fish can regulate feed intake to satisfy their energy metabolic requirements [34,35,36]. Nonetheless, when diets lack an essential nutrient, fish may consume more feed to meet the specific nutrient requirement [37]. In the present study, the DFI of juvenile dotted gizzard shad was significantly influenced by different CP levels. The highest DFI value was observed in fish fed the lowest dietary CP diet (CP1), which was significantly higher than that in CP3, CP4, and CP5 diets. A general enhancing trend in DFI with descending dietary protein levels has also been observed in *L. macrochirus* [6], *E. akaara* [7], and *Eleginops maclovinus* [37]. It is well known that fish require adequate protein intake to obtain benign growth performance. However, when fish are fed diets with insufficient protein inclusion, a high proportion of protein in diets may be applied to nitrogen deposition [3]. The results of this study support the hypothesis that the values of PER and NR increase significantly with the reduction of dietary CP levels. A similar result also appeared in the study of *L. macrochirus* [6]. Although fish fed with the CP1 diet had excellent DFI, PER, and NR compared with those fed with higher levels of dietary protein (CP2, CP3, CP4), obvious reductions in WG, DNI, and DNG were also observed in the CP1 diet. These data suggest that the rise of DFI, NR, and PER of this species might not be enough to obtain a sufficient amount of protein in a low-protein diet. Similar results have been reported for *Megalobrama terminalis* [38]. Therefore, the lack of dietary protein may be the main reason for the phenomenon of fish exhibiting poor growth and reduced feed utilization [39].

Protein to energy (P/E) ratio plays a considerable role in establishing a formulated feed, which may affect growth performance and feed efficiency of fish [6,7,40]. There is a strong view that P/E ratio is a more reasonable way of expressing protein requirements than dietary crude “protein requirements” [40]. In this study, the addition of corn starch was used to balance the energy of the diets with P/E ratios ranging from 11.69 mg kJ^−1^ to 24.15 mg kJ^−1^. After the experiment, dotted gizzard shad fed 22.52% CP diet with P/E ratio of 11.69 mg kJ^−1^ showed poor growth performance and feed utilization, which may be attributed to the unbalanced P/E ratio of diets. Although the DFI value of the P1 diet is significantly higher than that of feed with higher levels of dietary protein (CP2, CP3, and CP4), nitrogen intake is still insufficient, and more lipid is ingested, which further exacerbates the imbalance of nutrients and affects the utilization of protein. On the other hand, fish cannot use excessive dietary protein for protein synthesis because a high content of dietary protein is usually used as energy fuel for metabolism [41]. In this study, fish fed the diet with P5 obtained the lowest WG and SGR values. The result may be ascribed to the excessive amino acid metabolism in the high proportion of dietary CP [42], which causes additional energy consumption through deamination and amino acid metabolism [27].

In this study, although the values of DEI and DLI in the CP1 diet were the highest, the lowest values of ER and LR were also observed in the CP1 diet. Sá et al. [37] reported that fish fed the low-protein diets (isoenergetic) had poor digestibility, and the increase of feed intake was probably to overcome the digestibility of low-protein diets. In this study, corn starch was used to balance energy, and cellulose made up for the insufficiency of the diets, leading to the lowest protein group (CP1) having the highest content of corn starch and cellulose. Usually, high content of cellulose may result in poor digestibility for fish [6,37]. In a sense, the imbalance of formula, or excessive intake of non-protein energy may be the important influencing factors of poor growth performance and feed utilization in fish fed low-protein diets. NR and PER could reflect the utilization of dietary protein [30]. In this study, DNI values in CP4 and CP5 diets were significantly higher than those in the CP2 diet. Although the ER and LR values of CP3, CP4, and CP5 diets were not significantly different from those of the CP2 diet, the NR value of the CP5 diet was significantly lower than that of the CP2 diet. On the other hand, the FCR value gradually increased as dietary protein level increased from 34.85% to 45.78%. The studies of *N. japonica* [39] and *L. macrochirus* [6] also showed that the high-protein diets had poor feed utilization. Moreover, the lowest PER value was observed in the CP5 diet; and the PER value of the CP5 diet was significantly lower than that of CP2, CP3, and CP4 diets. Similarly, the research of *Diplodus puntazzo* [34] and *M. terminalis* [38] also found the PER value decreased gradually with the increase of dietary CP level. Generally, high level of dietary protein contains insufficient non-protein energy, and excessive dietary protein is considered metabolized for energy or converted into lipid [39]. It may lead to a decrease in the efficiency of protein utilization, increase the metabolic load of the fish body, and even cause ammonia toxicity [30,31]. Therefore, it is necessary to avoid the high FCR and low PER caused by high protein content in fish diets, which leads to high feed cost, excessive nitrogen excretion, and even harm to fish.

For some fish, excessive dietary protein may be used as lipid deposits [29,38] or energy metabolism [9]. Compared with the CP3 diet, the dotted gizzard shad fed with the CP4 diet showed relatively low PER and NR values, whereas the LR value of the CP3 diet was higher than that of the P4 diet. Moreover, the crude lipid of the whole body in the CP4 diet is higher than that in the CP3 diet, which indicates that excessive dietary protein may not be used for protein deposition, but may be deposited as lipid. This is consistent with the studies of *T. rubripes* [27] and *Brachymystax lenok* [39]. However, some other studies also showed that the crude lipid of the whole body for *L. macrochirus* [6] and *E. akaara* [7] decreased significantly with the increase of dietary CP levels. The difference may be related to fish species or culture environment. There were no significant differences in the crude protein of the whole body among treatments, suggesting the deposition of body protein of juvenile dotted gizzard shad was not affected by the level of dietary CP. This is consistent with the study of *E. akaara* [7]. These observations may be attributed to the fact that the protein biological titer of fish with low-protein diets is apparently higher than that of fish with high-protein diets, so as to make up for the deficiency of dietary protein level and ensure the accumulation of body protein [41]. This hypothesis is consistent with the highest NR value of the lowest dietary protein level. In addition, there was no significant difference in the moisture of the whole body among CP treatments. This is similar to the results of *E. akaara* [7] and *T. rubripes* [27].

Digestive enzyme activity (including amylase and lipase) is considered as a predictor of potential feed utilization and growth differences of some fish [12,43]. Wang et al. [44] reported that *Nibea albiflora* fed the diet with 47% CP has significantly higher intestinal lipase activity than those fed 40% and 54% CP diets. Moreover, Ping et al. [45] reported that the activity of hepatopancreas amylase was increased to some extent, and then reduced as dietary CP levels continued to increase. These observations showed that the increase of dietary protein level in a certain range may promote the secretion of lipase and amylase, enhance the absorption of the digestive function of fish, and be beneficial to fish growth [46]. However, excessive or too low dietary protein may lead to the decrease of enzyme activity, which is not conducive to growth and health [47]. Similar results also appeared in our study, and the trend of lipase and amylase activities was generally consistent with the growth and feed utilization of juvenile *K. punctatus*.

The content of dietary protein can affect the activities of enzymes involved in amino acid catabolism [48]. Aminotransferase such as GPT could decompose amino acids and transfer amino groups to alpha-keto acids (reversible catalysis) [12]. The activity of GPT reflects the metabolic intensity of amino acids and can be used as a reference index to evaluate the nutrition and health of fish [47]. Zhang et al. [49] reported that *Channa maculata* ♀ × *Channa argus* ♂ fed the diet with 46% CP showed significantly higher plasma GPT activity than those fed 34% CP diet. Moreover, the activity of GPT is considered an indicator of proper liver function, and high GPT activity generally indicates a weakening or impairment of normal liver function [27,47]. In this study, GPT activity increased significantly as dietary CP levels increased from 22.52% to 34.85%, which may indicate the amino acid metabolism of *K. punctatus* was affected. The mechanism of dietary CP level on amino acid metabolism of different fish species still needs further study.

In conclusion, this study found that the suitable levels of dietary protein for juvenile *K. punctatus* is recommended to be 31.75–33.82%, based on WG and FCR when fish meal was used as the sole protein. Inadequate or excessive dietary protein levels are unfavorable for fish growth and feed utilization. In addition, higher dietary protein levels also alter the digestive enzyme activities and metabolism of amino acids.

## Figures and Tables

**Table 1:** Formulation and composition of experimental diets (% as dry matter basis).

Ingredients (g 100 g^−1^)	Dietary Crude Protein (CP) Levels (%)				
	22.52 (CP1)	28.69 (CP2)	34.85 (CP3)	38.84 (CP4)	45.78 (CP5)
Fish meal ^1^	29.43	38.25	47.08	55.91	64.74
Corn starch ^2^	34.02	28.02	22.02	16.02	10.02
Fish oil ^3^	3.85	3.24	2.63	2.02	1.41
Soybean oil ^4^	2.94	2.94	2.94	2.94	2.94
Vitamin mix ^5^	2.50	2.50	2.50	2.50	2.50
Mineral mix ^6^	1.50	1.50	1.50	1.50	1.50
Vitamin C	0.50	0.50	0.50	0.50	0.50
Choline chloride	0.30	0.30	0.30	0.30	0.30
Sodium alginate	4.00	4.00	4.00	4.00	4.00
Cellulose	20.96	18.74	16.53	14.31	12.09
Total	100.00	100.00	100.00	100.00	100.00
Proximate composition (g 100 g^−1^ dry matter)					
Moisture	4.10	4.18	3.99	5.04	4.67
Crude protein	22.52	28.69	34.85	38.84	45.78
Crude lipid	9.03	8.38	8.37	8.55	8.88
Gross energy (kJ g^−1^)	19.26	18.77	18.91	18.91	18.96
Protein to energy ratio (mg kJ^−1^)	11.69	15.29	18.43	20.54	24.15

^1^ Imported from Trident Seafoods Corporation, Seattle, USA. ^2^ Obtained from Sanzhenzhai Foodstuff Co., Ltd., China. ^3^ Obtained from Zhejiang Industrial Group Co., Ltd., China. ^4^ Obtained from Jia Li Food Co., Ltd., China. ^5^ Vitamin (g kg^−1^ premix) premix consisted of the following ingredients: menadione (4.00); riboflavin (5.00); inositol (200.00); biotin (0.60); folic acid (1.50); cyanocobalamin (0.01); D-Ca pantothenate (10.00); nicotinic acid (20.00); pyridoxine hydrochloride (4.00); thiamin hydrochloride (5.00); tocopherol (40.00); axerophthol (5.00); Vitamin D (4.80); α-cellulose (700.09). ^6^ Mineral (g kg^−1^ premix) premix consisted the following ingredients: MgSO_4_·7H_2_O (90.43); Ca(H_2_PO_4_)_2_ (122.87); KI (0.02); KH_2_PO_4_ (42.03); FeSO_4_·7H_2_0 (19.73); CuSO_4_·5H_2_O (0.34); NaCl (32.33); KCl (65.75); CoCl_2_·6H_2_O (0.79); ZnSO_4_·7H_2_O (8.44); ferric citrate (38.26); K_2_SO_4_ (163.83); C_6_H_10_CaO_6_·5H_2_O (683.62); MnSO_4_·H_2_O (0.37).

**Table 2:** Growth performance and feed utilization of juvenile *K. punctatus* fed diets containing different CP levels.

Items	Dietary CP Levels (%)				
	22.52 (CP1)	28.69 (CP2)	34.85 (CP3)	38.84 (CP4)	45.78 (CP5)
IBW (g fish^−1^) ^1^	3.51 ± 0.06	3.46 ± 0.13	3.48 ± 0.11	3.68 ± 0.16	3.91 ± 0.15
FBW (g fish^−1^) ^2^	9.77 ± 0.57	9.96 ± 0.64	9.94 ± 0.66	10.70 ± 0.82	10.70 ± 0.39
Survival (%)	96.97 ± 5.25	95.45 ± 4.55	93.94 ± 6.94	92.42 ± 6.94	92.42 ± 13.12
WG (%) ^3^	177.96 ± 11.84	185.41 ± 7.94	187.92 ± 25.74	191.12 ± 21.95	173.93 ± 10.64
SGR (% day^−1^) ^4^	1.82 ± 0.08	1.88 ± 0.05	1.87 ± 0.16	1.90 ± 0.14	1.80 ± 0.07
FCR ^5^	1.97 ± 0.27	1.70 ± 0.04	1.54 ± 0.21	1.54 ± 0.20	1.73 ± 0.12
DFI (g 100 g fish^−1^ day^−1^) ^6^	4.92 ± 0.71 ^b^	4.42 ± 0.96 ^ab^	3.92 ± 0.14 ^a^	3.83 ± 0.19 ^a^	3.85 ± 0.15 ^a^
PER ^7^	2.18 ± 0.19 ^c^	1.97 ± 0.44 ^bc^	1.81 ± 0.24 ^b^	1.60 ± 0.20 ^b^	1.20 ± 0.81 ^a^

^1^ Initial body weight; ^2^ final body weight; ^3^ weight gain; ^4^ specific growth rate; ^5^ feed conversion rate; ^6^ daily feed intake; ^7^ protein efficiency ratio. Values are the means of three replicates; Means in the same row with different superscripts are significantly different (*p* < 0.05).

**Table 3:** Nitrogen, energy, and lipid utilization by juvenile *K. punctatus* fed diets containing different CP levels.

Items	Dietary CP Levels (%)				
	22.52 (CP1)	28.69 (CP2)	34.85 (CP3)	38.84 (CP4)	45.78 (CP5)
Nitrogen					
DNI (g kg^−1^ ABW^−1^ day^−1^) ^1^	1.51 ± 0.19 ^a^	1.73 ± 0.01 ^b^	1.89 ± 0.02 ^bc^	2.03 ± 0.05 ^c^	2.43 ± 0.07 ^d^
DNG (g kg^−1^ ABW^−1^ day^−1^) ^2^	0.39 ± 0.05	0.44 ± 0.01	0.44 ± 0.03	0.40 ± 0.09	0.41 ± 0.04
NR (%) ^3^	26.60 ± 6.24 ^b^	25.50 ± 0.40 ^b^	23.08 ± 1.71 ^ab^	19.93 ± 4.92 ^ab^	17.09 ± 1.92 ^a^
Energy					
DEI (kJ kg^−1^ ABW^−1^ day^−1^) ^4^	8.03 ± 1.041 ^b^	7.08 ± 0.02 ^a^	6.41 ± 0.08 ^a^	6.16 ± 0.15 ^a^	6.29 ± 0.19 ^a^
DEG (kJ kg^−1^ ABW^−1^ day^−1^) ^5^	3.89 ± 0.24	4.03 ± 0.18	3.80 ± 0.69	4.11 ± 0.17	3.91 ± 0.10
ER (%) ^6^	48.82 ± 5.87 ^a^	56.86 ± 2.80 ^ab^	59.36 ± 10.88 ^ab^	66.80 ± 4.31 ^b^	62.15 ± 0.30 ^b^
Lipid					
DLI (g kg^−1^ ABW^−1^ day^−1^) ^7^	3.77 ± 0.49 ^b^	3.16 ± 0.01 ^a^	2.84 ± 0.03 ^a^	2.79 ± 0.07 ^a^	2.95 ± 0.09 ^a^
DLG (g kg^−1^ ABW^−1^ day^−1^) ^8^	1.75 ± 0.38	2.03 ± 0.12	1.71 ± 0.21	1.96 ± 0.44	1.83 ± 0.07
LR (%) ^9^	47.82 ± 5.06 ^a^	64.08 ± 4.03 ^ab^	60.15 ± 7.15 ^ab^	70.52 ± 7.46 ^b^	62.03 ± 1.64 ^ab^

^1^ Daily nitrogen intake; ^2^ daily nitrogen gain; ^3^ nitrogen retention; ^4^ daily energy intake; ^5^ daily energy gain; ^6^ energy retention; ^7^ daily lipid intake; ^8^ daily lipid gain; ^9^ lipid retention. Values are the means of three replicates; Means in the same row with different superscripts are significantly different (*p* < 0.05).

**Table 4:** Whole body composition of juvenile *K. punctatus* fed diets containing different CP levels.

Items (%)	Dietary CP Levels (%)				
	22.52 (CP1)	28.69 (CP2)	34.85 (CP3)	38.84 (CP4)	45.78 (CP5)
Moisture	71.93 ± 0.85	70.54 ± 0.56	71.25 ± 1.44	70.86 ± 1.07	71.04 ± 0.97
Crude protein	14.10 ± 1.21	15.03 ± 0.39	14.97 ± 0.12	14.01 ± 1.70	14.75 ± 0.78
Crude lipid	9.76 ± 1.49	10.63 ± 0.36	9.46 ± 0.37	10.30 ± 1.30	10.11 ± 0.14

## Data Availability

The data presented in this study are available on request from the corresponding author. The data are not publicly available due to privacy.
